# The influence of microbial colonization on inflammatory versus pro-healing trajectories in combat extremity wounds

**DOI:** 10.1038/s41598-024-52479-5

**Published:** 2024-03-04

**Authors:** Seth A. Schobel, Eric R. Gann, Desiree Unselt, Scott F. Grey, Felipe A. Lisboa, Meenu M. Upadhyay, Michael Rouse, Simon Tallowin, Nicholas A. Be, Xijun Zhang, Clifton L. Dalgard, Matthew D. Wilkerson, Milos Hauskrecht, Stephen F. Badylak, Ruben Zamora, Yoram Vodovotz, Benjamin K. Potter, Thomas A. Davis, Eric A. Elster

**Affiliations:** 1 Department of Surgery, Uniformed Services University of the Health Sciences, Bethesda, MD USA; 2 Uniformed Services University (USU) Surgical Critical Care Initiative (SC2i), Bethesda, MD USA; 3 Henry M. Jackson Foundation for the Advancement of Military Medicine, Inc., Bethesda, MD USA; 4 Academic Department of Military Surgery and Trauma, Royal Centre for Defence Medicine, Birmingham, UK; 5 Physical and Life Sciences Directorate, Lawrence Livermore National Laboratory, Livermore, CA USA; 6 Uniformed Services University (USU) The American Genome Center (TAGC), Bethesda, MD USA; 7 Department of Anatomy, Physiology & Genetics, Uniformed Services University of the Health Sciences, Bethesda, MD USA; 8 Department of Computer Science, University of Pittsburgh, Pittsburgh, PA USA; 9 Department of Surgery, University of Pittsburgh, Pittsburgh, PA USA; 10 Center for Inflammation and Regeneration Modeling, McGowan Institute for Regenerative Medicine, University of Pittsburgh, Pittsburgh, PA USA; 11 Department of Bioengineering, University of Pittsburgh, Pittsburgh, PA USA; 12 Walter Reed National Military Medical Center, Bethesda, MD USA; 13 Present Address: Q2 Solutions, Durham, NC USA

**Keywords:** Inflammation, Transcriptomics

## Abstract

A combination of improved body armor, medical transportation, and treatment has led to the increased survival of warfighters from combat extremity injuries predominantly caused by blasts in modern conflicts. Despite advances, a high rate of complications such as wound infections, wound failure, amputations, and a decreased quality of life exist. To study the molecular underpinnings of wound failure, wound tissue biopsies from combat extremity injuries had RNA extracted and sequenced. Wounds were classified by colonization (colonized vs. non-colonized) and outcome (healed vs. failed) status. Differences in gene expression were investigated between timepoints at a gene level, and longitudinally by multi-gene networks, inferred proportions of immune cells, and expression of healing-related functions. Differences between wound outcomes in colonized wounds were more apparent than in non-colonized wounds. Colonized/healed wounds appeared able to mount an adaptive immune response to infection and progress beyond the inflammatory stage of healing, while colonized/failed wounds did not. Although, both colonized and non-colonized failed wounds showed increasing inferred immune and inflammatory programs, non-colonized/failed wounds progressed beyond the inflammatory stage, suggesting different mechanisms of failure dependent on colonization status. Overall, these data reveal gene expression profile differences in healing wounds that may be utilized to improve clinical treatment paradigms.

## Introduction

Modern warfare is ever-evolving. The recently concluded counter-insurgency missions in Iraq and Afghanistan for example were more asymmetrical^1^, characterized by widespread use of improvised explosive devices (IEDs). This led to extremity blast injuries replacing gun-shot wounds as the dominant characteristic injury^2–4^. This is not unique to past conflicts either, but appears to be the characteristic injury of modern warfare in general. In the ongoing war in Ukraine, blast injuries make up a significant portion of injuries involving not only warfighters^5^, but also civilians^6^. Advances in protective equipment, early evacuation, and treatment have dramatically improved survival from this form of combat trauma^2,7, 8^, although mortality remains prevalent^3,7^. Current surgical approaches to mitigate further complications of extremity injuries involve delayed wound closure. This treatment involves serial debridement operations occurring every 2–3 days, according to military developed protocols^9^, to remove necrotic and non-viable tissue and, in association with antibiotic treatment, to manage infections prior to definitive closure. Despite these advances, treatment can still be improved as the rate of complications, such as wound failure, as well as the lower quality of life post injury remain prevalent^4,10^. This is in part due to the complexity of wound healing, which must progress through multiple coordinated processes regulated by various cytokines, chemokines, and immune cell types. The coordinated healing process progresses from biological functions involved in prevention of blood loss and removal of necrotic tissue and defense from pathogens, to the generation of extracellular matrix, new blood vessels, nerves, and finally scar tissue^11^. Utilizing proteomic, gene expression, and metabolomic approaches in both clinical samples and animal model studies^12–15^, much has been learned about signatures of properly healing wounds compared to those which fail.

One important driver of wound failure is microbial colonization, or the growth of microorganisms (i.e., bacteria or fungi) in and on the wound bed. The wound bed can be introduced to these microorganisms either upon initial insult or in the hospital setting. Microbial colonization, and subsequent infections of the wounds due to microbial overgrowth has a high prevalence in extremity blast injuries^10^. Microbial colonization is associated with an increased inflammatory response both locally and systemically^14,16^. Microbial colonization has been shown to cause an activation of an exaggerated immune responses through various Toll-like receptors leading to the production of pro-inflammatory cytokines which in turn recruits various immune cells leading to prolonged inflammation^17^. This modulation of the inflammatory state leads to a dysregulation of downstream healing processes^18^. Further, infected wounds, such as those with either bacterial, or invasive fungal infections, frequently have more complications and worse outcomes, requiring additional surgeries or even amputation^19^. Our group has previously investigated the influence of microbial colonization in combat casualty extremity wounds using metagenomic sequencing^20^. It was found that there was not a single defining microbial profile that was associated with failed wounds, but trends such as *Pseudomonas* and *Acinetobacter* colonization were seen at higher prevalence, as well as lower observed alpha and beta diversities.

Despite the growing wealth of knowledge on the wound healing process, and what can potentially lead to wound failure, the timing of wound closure is based on clinical examinations of visual signs of healing determined by surgeons^9,12, 13^. Timing of surgical wound closure is paramount, premature definitive surgery can lead to infection, dehiscence, or failure, while unnecessary debridement surgeries increase risks of additional nosocomial complications, delays healing, and increases costs^13^. One potential solution to improve the current methods is to develop clinical decision support tools or novel therapies. Our group has previously used circulating serum biomarker data^12^, gene expression data^13^, and microbial metagenomic signatures^20^, as features in machine learning models that enable the prediction of wound failure. To further understand the mechanisms of the wound healing process and predict novel biomarkers, this study utilized RNA sequencing of wound tissue biopsies collected longitudinally during treatment of U.S. military service members who sustained combat extremity injuries. The overall goal of this study was to further understand the biological underpinnings of wound failure in both colonized and non-colonized wounds (i.e., those with detectable viable bacteria vs those without). We hypothesized that differences in inflammatory versus progressive pro-healing responses lead to varied healing outcomes both in the presence and absence of mitigating microbial colonizers. Gene expression profiles were analyzed to identify differences in gene expression, dynamic multi-gene networks, and longitudinal profiles of wound healing functions to determine differences in functional profiles temporally. These data suggested that different mechanisms of wound failure existed in colonized wounds compared to non-colonized wounds.

## Methods

### Retrospective study design

These samples were previously collected from U.S. service members who were wounded during combat in Iraq or Afghanistan in compliance with all federal regulations governing the protection of human subjects and informed consent that was approved by the Walter Reed National Military Medical Center Institutional Review Board (Walter Reed National Military Medical Center Institutional Review Board protocol #352334). Written informed consent was obtained from all subjects in this study. All methods were performed according to relevant regulations, and experimental protocols were carried out in accordance with guidelines from relevant institutional committees. Participants were enrolled between 2007 and 2012. Inclusion criteria were defined as any service individual that sustained at least one extremity injury resulting in a wound > 75 cm^2^, treated with negative wound therapy, and undergoing multiple surgical debridements and delayed wound closure. A maximum of 3 extremity wounds were evaluated per individual. Exclusion criteria included patients with immune disorders. Surgical debridements, followed by vacuum-assisted negative pressure wound therapy, were repeated every 48 to 72 h until definitive wound closure. The timing of wound closure was determined by attending surgeons. After definitive wound closure, if a wound experienced infection, dehiscence, or partial loss of a skin graft or flap, which required additional surgical procedures, the wound healing outcome was categorized as a wound failure. This was evaluated for a period of 30 days after wound closure^9,12, 13^.

### Sample collection and processing

During surgery, after wound debridement procedures were completed, viable (non-necrotic) wound biopsy tissue (1 cm^3^) was collected from the center of the wound, preserved in RNAlater, and stored at − 80 °C until processing. Wound tissue biopsy samples were examined from 21 wounds in 18 military patients. The samples were chosen from these patients as they underwent multiple debridements, and there were sufficient available samples for subsequent studies.

### Quantitative bacteriology

Tissue biopsy samples were homogenized with a sterile disposable tissue grinder and diluted 1:10 (wt/vol) in fastidious broth growth medium. Diluted homogenates were plated on Sheep’s blood agar and MacConkey agar plates in triplicate and incubated overnight at 37 °C. Following incubation, enumeration of bacterial colonies was performed and the number of colony-forming units (CFUs) per gram of tissue was calculated. Phenotypic identification of colonies was accomplished using the Phoenix automated bacterial identification system (Becton, Dickinson and Company, Franklin Lakes, United States).

### RNA extraction and purification

To extract RNA, 25–30 mg of tissue was homogenized in a sterile RNase- and DNase-free Eppendorf tube containing 300 μL lysis buffer (RNeasy Mini Kit, Qiagen, Hilden, Germany). Total RNA was isolated using the RNeasy Mini Kit (Qiagen, Hilden, Germany) according to the manufacturer’s instructions. RNA yield was determined using a NanoDrop ND-8000 spectrophotometer (Thermo Fisher Scientific, Waltham, United States). Quality and integrity of total RNA was further assessed using an Agilent 2100 Bioanalyzer (Agilent Technologies, Santa Clara, United States).

### RNA sequencing, processing, and differential expression analysis

To quantify genome-wide RNA gene expression patterns in these wounds, stranded total RNA libraries of 67 samples were sequenced using the NextSeq 500 platform (Illumina, San Diego, United States). Sequencing reads were aligned to the human reference genome (hg38) using Mapsplice^21^. Three libraries were removed due to low quality and therefore the final dataset included 64 transcriptomic datasets. Transcript quantification was performed using HTSeq^22^. Samples were further defined in days post-injury and classified in groups that were: 3–5 days, 6 days, 7 days, 8–10 days, 11–12 days, 13–16 days, and greater than 17 days post-injury. These timepoint groupings were chosen to balance obtained samples between healed and failed wounds for differential gene expression analysis. Differential gene expression analysis was performed using DESeq2^23^ (Supplementary Dataset S1).

### Concordance analyses between previous qRT-PCR study and current study

Concordance between gene expression profiles determined by RNA sequencing were compared to those identified using quantitative reverse transcriptase polymerase chain reaction (qRT-PCR) published previously^13^. Both studies analyzed gene expression in the same wounds, though separate RNA extractions were performed from different portions of the same tissue biopsy. To examine correlations between expression of specific cytokines and wound healing markers derived from the previous qRT-PCR study and RNA sequencing data from this study, only matched samples (n = 55) and genes (n = 148) were used. Normalization was performed across genes and across samples using median center (Supplementary Dataset S2, Supplementary Dataset S3). Spearman’s rank correlation coefficient ($\rho$) was calculated using normalized TPM values and normalized threshold cycles for each gene (Supplementary Dataset S4).

### Dynamic network analysis

Dynamic network analysis (DyNA)^24^ was carried out to define the central network nodes as a function of both time and patient sub-group. Using the expression data for each wound outcome (healed vs. failed), networks were created over two consecutive periods (d4–5, d5–6, etc.) using MATLAB software; data were not binned. Connections ([edges], or number of trajectories of transcripts that move in parallel) were created if the Pearson correlation coefficient between any two nodes (genes) at the same time interval was greater or equal to a threshold of an absolute value of 0.99. The network complexity for each time interval was calculated using the following formula: Sum (N_1_ + N_2_ + ⋯ + N_n_)/(n − 1), where N represents the number of connections for each genes, and n is the total number of genes analyzed.

### Gene ontology and deconvolution of RNA-Seq data analysis

Differentially expressed genes (DEGs) sets were analyzed for enrichment in Gene Ontoloies (GO) using g:Profiler (adjusted *p* value < 0.05)^25^ (Supplementary Dataset S5). GO terms of interest were manually selected, and grouped into 19 general categories (actin, adhesion, calcium, collagen, cytokine, extracellular matrix, immune system, inflammation, leukocytes, lymphocytes, metal ion transport and homeostasis, muscle, myosin, neutrophils, oxygen transport and hemoglobin, peptidases/proteases, Redox, T cells, translation) (Supplementary Dataset S6, Supplementary Dataset S7). These categories were of interest as they pertain to immune functions, healing functions, muscle, and host defense, and therefore are relevant to the wounds from this study. To determine changes over time within grouped GO terms, the DESeq2 normalized read counts for each gene within that category were summed for each sample (Supplementary Dataset S8). This was done by converting all locus tags to ENSEMBL IDs using tables downloaded from the UCSC genome browser^26^, and then summing those normalized read counts of ENSEMBL IDs associated with that GO term as determined by BioMart (Ensembl release 104)^27^. Deconvolution of the gene expression data was performed using quanTIseq^28^, which estimated the proportion of 10 immune cell types and ‘other’ cell types that do not fit into one of the defined immune cell types using the TPM counts (Supplementary Dataset S9).

Summed normalized read counts for GO functional category analysis and inferred proportions of immune cell types performed by quanTIseq were plotted over time, with linear models generated for the data for each of the four wound colonization status/outcome groups (Colonized/healed, Non-colonized/healed, Colonized/failed, and Non-colonized/failed). Spearman’s rank correlation coefficients ($\rho$) were calculated between the summed normalized read counts or inferred proportions of immune cell types and time post injury for each of the four wound colonization status/outcome groups (Supplementary Dataset S10). A matrix of these $\rho$ values were used to perform Principal Component Analysis in R.

## Results

### Combat casualty cohort and analysis strategy

**Figure 1:**
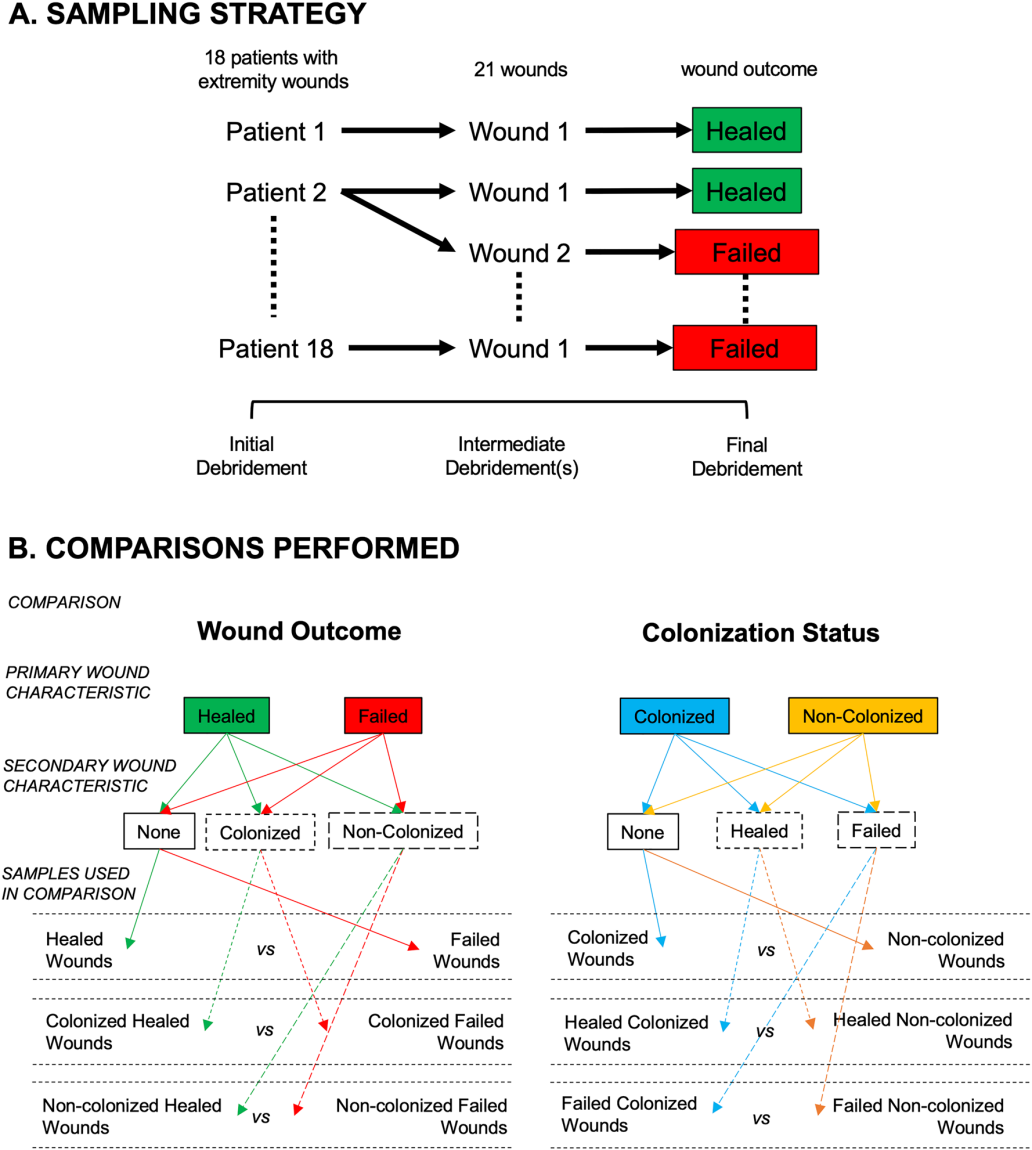
Study overview describing (**A**) the sampling strategy and (**B**) the comparisons performed for the differential gene expression analysis.

This military cohort has been described previously^9,12, 13^, and consists of 73 injured U.S. military service members with 116 wounds that underwent multiple surgical debridement procedures prior to definitive closure (Tables 1, 2). Wounds resulted from penetrating injuries in isolation or in combination with blunt-force trauma via gunshot wounds, crush or blast injuries. The primary outcome monitored was wound healing outcome (failed vs. healed) (Supplementary Table S1). A subset of samples were selected for sequencing, with the final dataset that passed quality control consisting of 64 transcriptomic libraries from 21 wounds in 18 patients (Fig. 1A, Tables 1, 2). This included samples from the same wound, at different debridements, over the course of treatment. The subset of patients selected for sequencing differ significantly from the entire cohort with a greater number of wounds (p = 0.035), and longer stays in the hospital (p = 0.008) (Table 1). In sequenced healed wounds, there were significant differences compared to sequenced failed wounds in the following variables: a greater number of debridements (p = 0.004), a later time (days post-injury) of wound closure (p = 0.002), a smaller wound volume (p = 0.019), and fewer associated vascular injuries in healed wounds (p = 0.016) (Table 2). The microbial load was also determined for each wound using culture-based techniques to determine CFUs/g tissue. In total, 19/64 tissue samples were collected from wounds that had viable bacterial growth at the time of sampling and were considered colonized wounds (Supplementary Table S1). Wounds that did not have viable bacterial growth were considered non-colonized wounds.

**Table 1:** Distribution of patients in the entire study and those used for RNA sequencing.

Patient (number (%) or median (IQR))	Whole study	RNASeq subset	p value
	n = 73	n = 18	
Age (years)	22.0 (20.0, 24.0)	21.5 (20.0, 23.8)	0.411
Gender			
Male	73.0 (100.0)	18.0 (100.0)	1.000
Body Mass Index (BMI)	25.1 (23.1, 26.8)	25.1 (22.7, 27.4)	0.857
Combat theater			0.637
Operation enduring freedom	43.0 (58.9)	10.0 (55.6)	
Operation Iraqi freedom	30.0 (41.1)	8.0 (44.4)	
Injury severity score (ISS)	16.0 (10.0, 22.0)	20.0 (16.0, 36.2)	0.086
Hospital length of stay (days)	22.0 (15.0, 39.0)	38.0 (25.0, 50.5)	**0.008**
ICU stay	29.0 (39.7)	10.0 (55.6)	0.637
ICU length of stay (days)	5.0 (4.0, 7.0)	5.5 (4.25, 7.0)	
Mechanism of injury			
Blast	63.0 (86.3)	16.0 (88.9)	1.000
Crush	1.0 (1.4)	1.0 (5.6)	0.358
GSW	9.0 (12.3)	1.0 (5.6)	0.680
Number of wounds			**0.035**
1	38 (52.1)	6 (33.3)	
2	27 (37.0)	7 (38.9)	
3	8 (10.9)	5 (27.8)	

Significant values are in bold.

Values are numbers with the percentage or interquartile range noted. For continuous variables p values were calculated with t tests while those non-normally distributed the Mann–Whitney *U* test was performed. For categorical variables the chi-squared test was performed for those that had cell sizes greater than 5, while the Fisher’s exact test was used when cell sizes were less than 5.

**Table 2:** Distribution of wounds in the entire study and those used for RNA sequencing.

Wound (number (%) or median (IQR))	Whole study		p value	RNASeq subset		p value
	Failed (n = 26)	Healed (n = 90)		Failed (n = 11)	Healed (n = 10)	
Wound type						
Extremity amputations	1.0 (3.8)	14.0 (15.6)	0.185	–	4.0 (40.0)	**0.035**
Fasciotomy	7.0 (26.9)	17.0 (18.9)	** < 0.001**	4.0 (36.4)	–	0.090
Open fractures	2.0 (7.7)	10.0 (11.1)	1.000	1.0 (9.1)	2.0 (20.0)	0.586
Soft tissue injury	16.0 (61.5)	49.0 (54.4)	0.399	6.0 (54.5)	4.0 (40.0)	0.670
Wound location			0.236			0.214
Lower extremity	24.0 (92.3)	73.0 (81.1)		11.0 (100.0)	8.0 (80.0)	
Upper extremity	2.0 (7.7)	17.0 (18.9)		–	2.0 (20.0)	
Wound closure (days from injury)	11.0 (8.0, 12.8)	10.0 (8.0, 12.0)	0.152	12.0 (8.5, 13.5)	16.5 (13.0, 25.8)	**0.002**
Size of wound						
Surface area (cm^2^)	289.5 (225.0, 443.8)	205.0 (151.5, 347.5)	0.003	425.0 (261.0, 572.0)	330.0 (156.8, 436.0)	0.116
Volume (cm^3^)	2245.0 (891.0, 3236.3)	590.0 (222.0, 1465.5)	** < 0.001**	2970.0 (1377.0, 5107.5)	682.5 (221.3, 1862.0)	**0.019**
Number of Debridements	3.0 (2.0, 3.0)	3.0 (2.0, 4.0)	0.457	3.0 (3.0, 4.0)	6.0 (5.0, 8.0)	**0.004**

Significant values are in bold.

Values are numbers with the percentage or interquartile range noted. P values are calculated for the comparison between dehisced and healed wounds for the whole study or the subset of the study used for sequencing. For continuous variables p values were calculated with t tests while those non-normally distributed the Mann–Whitney *U* test was performed. For categorical variables the chi-squared test was performed for those that had cell sizes greater than 5, while the Fisher’s exact test was used when cell sizes were less than 5.

### Wound outcome, colonization status, and time post-injury were associated with the number of differentially expressed genes

Differential gene expression analysis was performed to determine the number of differentially expressed genes (DEGs) when comparing differences in wound outcome (healed vs. failed wounds) or colonization status (colonized vs. non-colonized wounds), for all, or subsets, of the samples (Fig. 1B). The number of DEGs in the various comparisons ranged from 0 to > 2000, both across, and within comparisons, at different time intervals (Table 3, Supplementary Dataset S1). For the wound outcome comparison, significantly more genes were expressed differentially (paired t-test, p = 0.015) in colonized wounds compared to non-colonized wounds when comparing the number of DEGs across all shared time interval comparisons. With regard to colonization status, the number of DEGs in healed wounds compared to failed wounds when comparing the number of DEGs across all shared time interval comparisons was not statistically significant overall (paired t-test, p = 0.089). However, the comparison in failed wounds had 1–3 orders of magnitude more DEGs than the comparison in healed wounds both overall, and at later time-intervals (11–12 and 13–16 days post-injury) (Table 3).

**Table 3:** Number of differentially expressed genes for the comparison of wound outcome and colonization status.

Comparison: wound outcome: failed v. healed wounds												
Wound subset	All wounds				Colonized wounds				Non-colonized wounds			
Time groupings	Samples (failed v. healed)	Total	Up	Down	Samples (failed v. healed)	Total	Up	Down	Samples (failed v. healed)	Total	Up	Down
All samples	33 v. 31	22	4	18	10 v. 9	426	331	95	23 v. 22	14	3	11
Days 3–5	6 v. 7	3	1	2	3 v. 0	–	–	–	3 v. 7	3	2	1
Day 6	4 v. 4	0	0	0	2 v. 3	252	135	117	2 v. 1	31	12	19
Day 7	4 v. 4	11	3	8	1 v. 0	–	–	–	3 v. 4	34	5	29
Days 8–10	5 v. 6	5	5	0	2 v. 0	–	–	–	4 v. 5	12	7	5
Days 11–12	5 v. 5	57	24	33	2 v. 2	727	387	340	3 v. 3	97	37	60
Days 13–16	4 v. 5	17	11	6	1 v. 3	553	395	158	3 v. 2	16	2	14
Days 17 +	5 v. 0	–	–	–	0 v. 0	–	–	–	5 v. 0	–	–	–

Colonization status: colonized v. non-colonized wounds												
Wound subset	All wounds				Healed wounds				Failed wounds			
Time groupings	Samples (colonized v. non-colonized)	Total	Up	Down	Samples (colonized v. non-colonized)	Total	Up	Down	Samples (colonized v. non-colonized)	Total	Up	Down
All Samples	19 v. 45	989	565	424	10 v. 23	2	1	1	9 v. 22	2050	1203	847
Days 3–5	3 v. 10	6	1	5	3 v. 3	26	15	11	0 v. 7	–	–	–
Day 6	5 v. 3	3	2	1	2 v. 2	223	55	168	3 v. 1	35	15	20
Day 7	1 v. 7	248	22	226	1 v. 3	546	49	497	0 v. 4	–	–	–
Days 8–10	2 v. 9	6	2	4	1 v. 4	28	4	24	1 v. 5	15	6	9
Days 11–12	4 v. 6	2039	1205	834	2 v. 3	148	106	42	2 v. 3	2380	1280	1100
Days 13–16	4 v. 5	1369	607	762	1 v. 3	42	9	33	3 v. 2	2489	1443	1046
Days 17 +	0 v. 5	–	–	–	0 v. 5	–	–	–	0 v. 0	–	–	–

Both comparisons are broken into various subsets of the entire dataset including the timepoints and colonization status (for the wound outcome comparisons) and wound outcome (for colonization status).

### Many enriched GO terms were shared between samples but the proportion and presence of functional categories varied

Functional enrichment analysis was performed on both DEGs sets (upregulated and downregulated) for each comparison performed to understand changes in expression of whole functions and pathways based on the DEG sets (Table 3, Fig. 1B). In total, 5445 GO terms were enriched (2965 from upregulated DEG sets; 2479 from downregulated DEG sets), with 1960 being distinct across all DEG sets. The number of GO terms enriched varied by more than two orders of magnitude across DEG sets (Supplementary Table S2, Supplementary Dataset S5).

536/1960 distinct GO terms were categorized into 19 functional categories of interest (Supplementary Dataset S6). In total 3240/5445 GO terms enriched across all DEG sets could be categorized (Supplementary Dataset S7). There was variability in the total number, and proportion, of enriched GO terms that could be categorized in a specific functional category within one DEG set (Supplementary Fig. 1, Supplementary Dataset S7). The proportions of enriched GO terms categorized into a specific GO functional category ranged from 0.71 to 1.40% for lymphocyte functions to 0.43–31.72% for muscle functions (Supplementary Dataset S7).

### Dynamic network analysis suggests a lower degree of coordination of gene expression in healed vs. failed wounds

**Figure 2:**
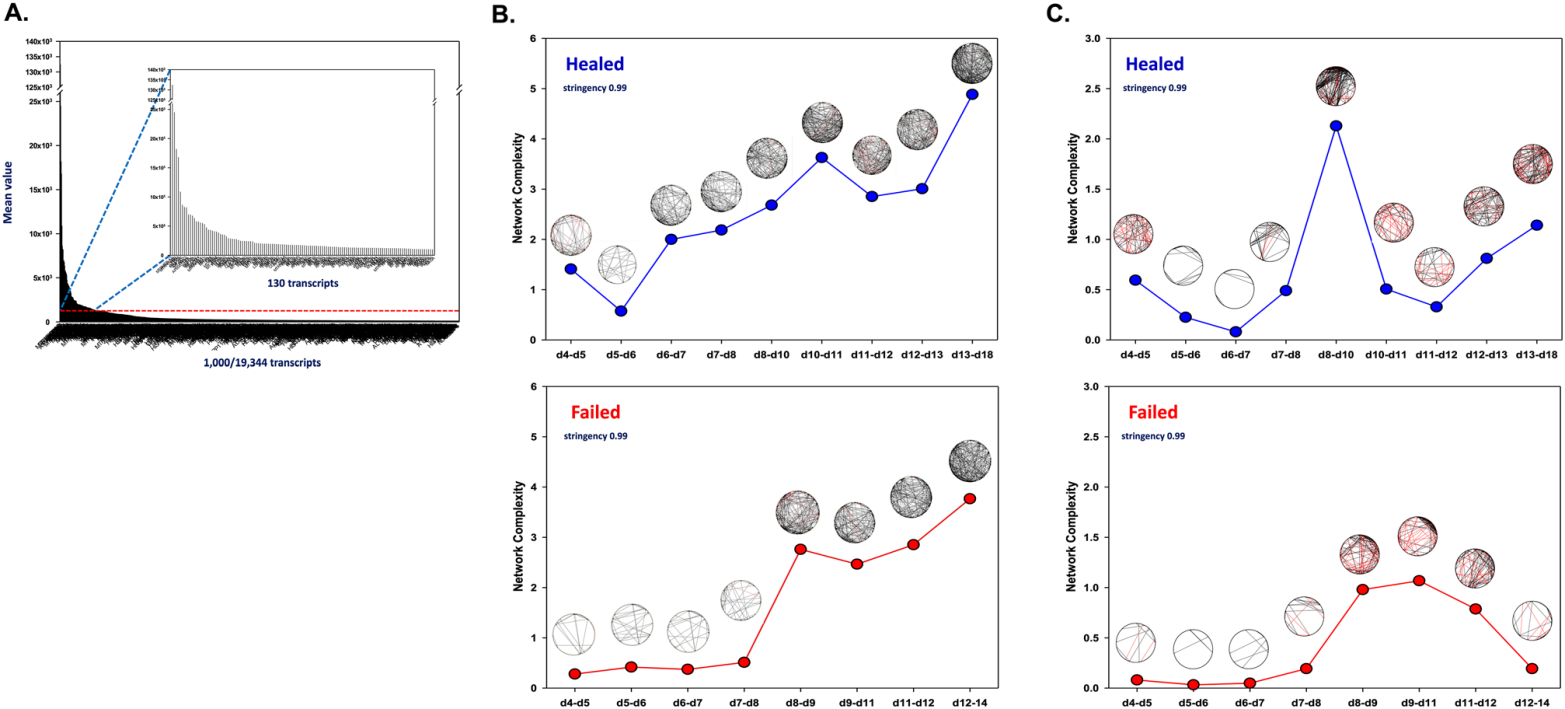
Dynamic Network Analysis (DyNA) reveals differences in the degree of coordination of gene expression based on wound outcome. (**A**) Unbiased gene down-selection of 130 transcripts based on mean expression value. (**B**) Changes in network complexity over the time groupings based on networks generated using the unbiased down-selection method for healed wounds and failed wounds (**A**). (**C**) Changes in network complexity over time groupings based on networks generated from genes down-selected using gene sets defined in KEGG and Reactome Databases^29,30^. Gene sets for both network analyses are described in Supplemental Dataset S11. The network complexity for each time grouping were calculated using the following formula: Sum (N_1_ + N_2_ + ⋯ + N_n_)/(n − 1), where N represents the number of connections for each gene, and n is the total number of genes analyzed. DyNA was carried out using a stringency of 0.99.

We next sought to define, using unbiased methods, the dynamic networks of gene expression in healed vs. failed wounds. First, we reduced the expression dataset by examining transcripts with values > 0 and a mean normalized TPM ≥ 1000, resulting in a total of 130 gene transcripts that were considered (Fig. 2A, Supplementary Dataset S11). These genes were used to analyze network evolution over time using Dynamic Network Analysis (DyNA)^24^ (Fig. 2B, Supplementary Table S3). This analysis suggested a lower degree of coordinated gene expression in failed wounds compared to healed wounds. However, given the large number of similar genes in the down-selected group of 130 (Supplementary Dataset S11), we repeated this analysis using a different approach to gene transcript down-selection, this time involving curated datasets. Genes were selected based on Gene Set Enrichment Analysis (GSEA) using EGSEA (with PADOG) to identify significant (adjusted p value < 0.05) gene sets from KEGG and Reactome^29–31^. This resulted in a list of 250 gene transcripts across all time points (Fig. 2C, Supplementary Table S3, Supplementary Dataset S11). This analysis showed a similar, though less pronounced, early, and lower degree of network complexity in failed wounds compared to healed wounds, and also suggested an earlier peak of organization in healed wounds compared to failed wounds.

### Correlations of GO functional categories to time post-injury varied based on wound group

**Figure 3:**
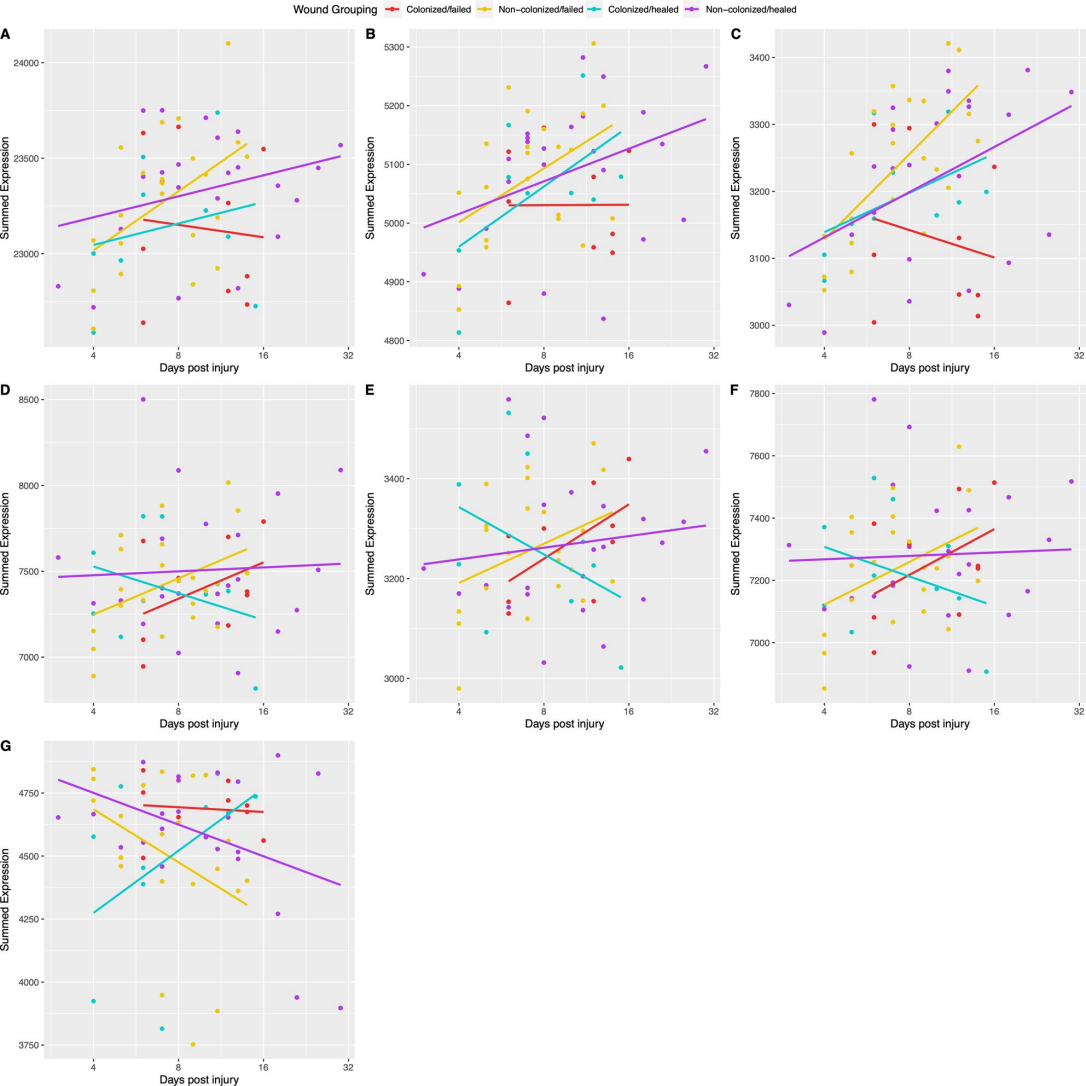
Expression profiles of GO functional categories over time reveal differences in function trajectories based on wound group. The expression profiles pertain to the following GO functional categories: (**A**) ECM, (**B**) Adhesion, (**C**) Collagen, (**D**) Immune system, (**E**) Inflammation, (**F**) Cytokines, and (**G**) Muscle. Each point represents the summed expression of all genes within that functional category in a particular transcriptomic library. Gene sets are described in Supplemental Dataset S8. The lines represent linear models generated comparing summed expression versus time for each wound group. Wound groups are denoted by color: with cyan being for colonized/healed wounds, purple being for non-colonized/healed wounds, red being for colonized/failed wounds, and yellow being for non-colonized/failed wounds.

The summed expression for each GO functional category was correlated to time post-injury for each of the four wound colonization status/outcome groups (Fig. 3, Supplementary Fig. S2, Fig. 5A, Supplementary Dataset S10). Colonized/healed wounds exhibited positive correlations of summed expression (ρ > 0.1) with time for 10 GO functional categories, flat/no correlation (0.1  >  $\rho$  > − 0.1) with time for 2 GO functional categories, and negative correlations ($\rho$  > − 0.1) for 7 GO functional categories (Fig. 5A, Supplementary Dataset S10). Non-colonized/healed wounds had positive correlations of summed expression with time for 6 GO functional categories, flat/no correlation with time for 9 GO functional categories, and negative correlations for 4 GO functional categories (Fig. 5A, Supplementary Dataset S10). Colonized/failed wounds had positive correlations of summed expression with time for 9 GO functional categories, flat/no correlation with time for 3 GO functional categories, and negative correlations for 6 GO functional categories (Fig. 5A, Supplementary Dataset S10). Non-colonized/failed wounds had positive correlations of summed expression with time for 12 GO functional categories, flat/no correlation with time for 2 GO functional categories, and negative correlations for 5 GO functional categories (Fig. 5A, Supplementary Dataset S10).

### Correlations of immune cell proportions to time post-injury varied based on wound group

**Figure 4:**
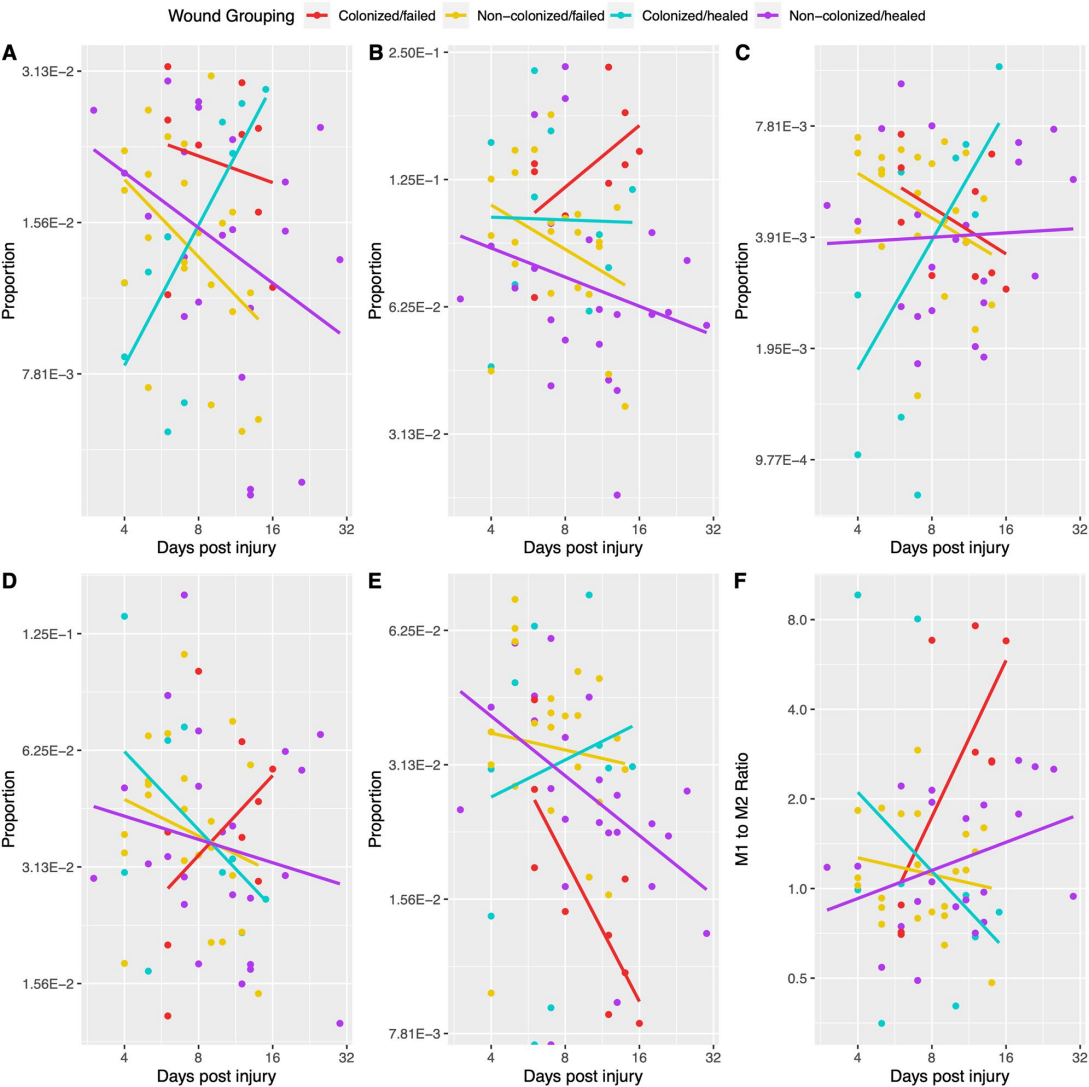
Predicted proportions of immune cell types over time reveal differences based on wound group. The proportion of immune cells were predicted using quanTIseq and the proportions of the following are shown: of (**A**) B cells, (**B**) Neutrophils, (**C**) Natural Killer cells, (**D**) M1 Macrophages, and M2 Macrophages (**E**), and (**F**) the ratio of M1 Macrophages to M2 macrophages. Each point represents the predicted proportion of that cell type in a particular transcriptomic library. Predicted proportions are described in Supplemental Dataset S9. The lines represent linear models generated comparing predicted proportions versus time for each wound group. Wound groups are denoted by color: with cyan being for colonized/healed wounds, purple being for non-colonized/healed wounds, red being for colonized/failed wounds, and yellow being for non-colonized/failed wounds.

**Figure 5:**
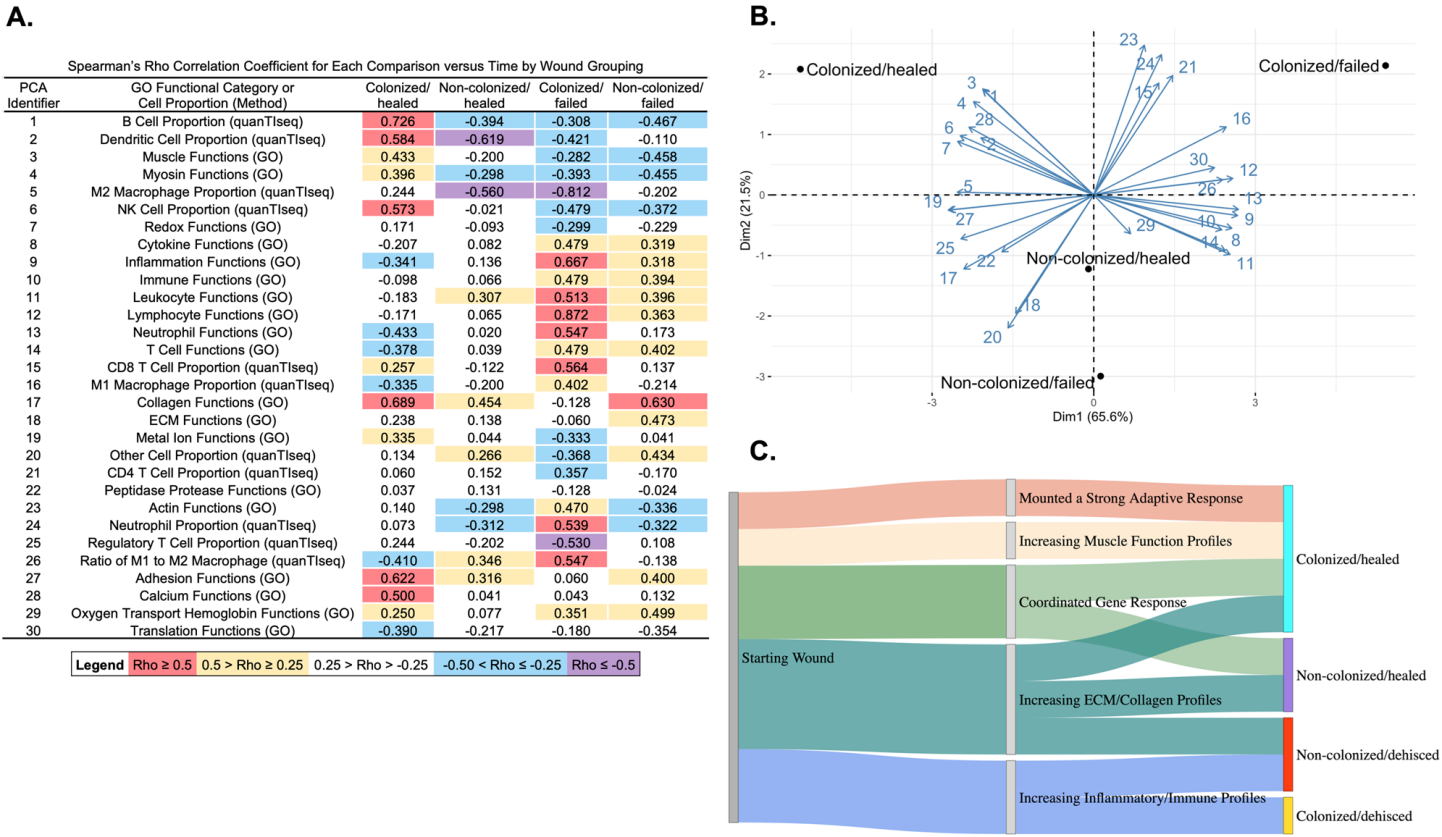
Principal Component Analysis shows distinct clustering of wound groupings based on analyses from this study. (**A**) Spearman’s rho correlation coefficients of linear models generated for GO functional categories expression profiles versus time and predicted immune cell proportions versus time for each wound group (Figs. 4,5). (**B**) Principal Component Analysis (PCA) generated from matrix of Spearman’s rho correlation coefficients (**A**). Each arrow is labeled with a number that corresponds to the PCA identifier in (**A**). (**C**) Schematic highlighting differences in the wound healing response based on wound grouping as determined from this study.

The proportions of 10 immune cells and “other” cells (those not categorized into the 10 immune cells) were inferred for each sample using the deconvolution method, quanTIseq, with all but monocytes being inferred to be present in at least one sample (Supplementary Dataset S9). Samples were grouped into the 4 wound groups and the proportion for each immune cell type was then correlated to time post-injury (Fig. 4, Supplementary Fig. S3, Fig. 5A, Supplementary Dataset S10). Colonized/healed wounds had positive correlations of inferred cell proportion with time for 6 immune cell types, flat/no correlation with time for 2 immune cell types, and negative correlations for 1 immune cell type (Fig. 5A, Supplementary Dataset S10). Non-colonized/healed wounds exhibited positive correlations of inferred cell proportions with time for 1 immune cell type, flat/no correlation with time for 1 immune cell type, and negative correlations for 7 immune cell types (Fig. 5A, Supplementary Dataset S10). Colonized/failed wounds had positive correlations of inferred cell proportions with time for 4 immune cell types, flat/no correlation with time for 0 immune cell types, and negative correlations for 5 immune cell types (Fig. 5A, Supplementary Dataset S10). Non-colonized/failed wounds had positive correlations of inferred cell proportions with time for 2 immune cell types, flat/no correlation with time for 0 immune cell types, and negative correlations for 7 immune cell types (Fig. 5A, Supplementary Dataset S10). The ratio of M1 to M2 macrophage proportion for each sample was also calculated (Fig. 4F, Fig. 5A, Supplementary Dataset S9). In colonized/failed wounds, this analysis suggested a larger increase in this ratio over time, whereas non-colonized/failed wounds and colonized/healed wounds decreased, suggesting an increasing inflammatory state.

### Principal component analysis reveals distinct groupings of wounds based on colonization status

A matrix of all of the $\rho$ values from the above correlations (Fig. 5A, Supplementary Dataset S10) was generated and used to perform a principal component analysis to determine how similar these four wound groups were in ordinate space (Fig. 5B). The first principal component explains more of the variance between the colonized groups (colonized/healed and colonized/failed), while the second principal component describes more of the variance between non-colonized samples (non-colonized/healed and non-colonized/failed). The range of the contributions each $\rho$ value contributed to the first principal component was between 0.322 for oxygen transport/hemoglobin GO functions to 5.04 for metal ion functions, while the contributions for each $\rho$ value were more varied in the second principal component (Supplementary Dataset S12).

### Cytokine and chemokine wound expression profiles are significantly correlated between qRT-PCR and RNA-Seq technologies

We compared our previously published cytokine and chemokine gene expression profiles done via qRT-PCR on this cohort^13^ to the current RNA sequencing data. When investigating 149 genes from both platforms, the mean Spearman correlation coefficient was 0.486, with *EGR1* and *PLA2G4A* having the greatest $\rho$ correlation coefficients at 0.829 and 0.804, respectively (Supplementary Dataset S4, Supplementary Fig. 4). Overall, gene expression was largely correlated between the two technologies, with 75/149 genes having Spearman’s $\rho$ correlation coefficients > 0.5 (Supplementary Dataset S4, Supplementary Fig. S4).

## Discussion

This study examined transcriptional profiles of tissue samples from combat extremity wounds sustained by warfighters during Operation Enduring Freedom and Operation Iraqi Freedom. Large volumetric muscle loss wounds are the characteristic injury type in modern warfare, from conflicts in Iraq and Afghanistan to those in Ukraine, and so providing insights into the biological underpinnings of wound failure is critical to the improvement of wound care moving forward. As there is a progression of coordinated responses over the healing process that are influenced by factors (i.e., microbial colonizers) that potentiate changes in the immune and inflammatory landscape^18,32^, samples were grouped based on wound outcome and colonization status to investigate the gene expression dynamics of wound healing functions and inferred immune cell proportions over time, although differences at distinct timepoints were noted as well. As these data are from a small number of patients and wounds, we performed a direct comparison of the gene expression of 149 genes determined by qRT-PCR over a decade ago and the gene expression determined by RNA sequencing conducted in this study. There was good correlation between the gene expression data across all comparable samples providing additional support for the exploratory results inferred from this study. This is strengthened further as similar conclusions as those discussed below have been drawn from the same patient cohort in previous studies^9,12, 13^.

There are many potential paths that any large wound can follow over the course of the healing process, below we summarize differences determined in this study. There were more pronounced differences between healed and failed wounds in colonized wounds compared to non-colonized wounds. This was seen in the differential expression analysis and with the profiles over time. Colonized/healed wounds had an increase in functional categories over time pertaining to muscle activity, ECM, collagen, and adhesion, while an observed decrease in functional categories pertaining to the immune system and the inflammatory response. These wounds also had increasing proportions of inferred B cells and Natural Killer cells over time, while a decreasing M1 to M2 macrophage ratio. These data provide support for the hypothesis that colonized/healed wounds were able to transition from the inflammatory stage (decreasing immune/inflammatory functional categories) to the proliferative stage (increasing ECM, collagen, and adhesion functional categories) by mounting an appropriate adaptive and cellular response (increasing B cell and NK cell proportions). This is supported further as the transition from a pro-inflammatory M1 macrophage state to an anti-inflammatory M2 macrophage state is critical in the resolution of the inflammatory stage and the promotion of fibrosis and scar tissue formation in wound healing^18^. In contrast, colonized/failed wounds were observed to have the inverse profile with increasing immune and inflammatory functional categories and decreasing/non-changing ECM, collagen, and adhesion functional categories, supporting the hypothesis that these wounds were unable to progress beyond the inflammatory stage of healing. The inferred proportions of immune cells for the colonized/failed wounds support an exaggerated response promoting inflammation with decreasing B cell and Natural Killer cell proportions over time but with increasing neutrophils proportions and M1/M2 macrophage ratio. Neutrophils have been shown to function primarily in the inflammatory phase of wound healing^18^, and without the clearance of neutrophils a prolonged inflammatory state can occur^32^, further supporting this hypothesis. Healed wounds also had more interconnected networks compared to failed wounds based on the dynamic network analysis providing support for a more coordinated response of these various processes.

The differences between the non-colonized wound groups were not as apparent in these data. Both non-colonized/healed wounds and non-colonized/failed wounds appeared to progress beyond the inflammation stage, as functional categories relating to ECM development increased over time. Non-colonized/failed wounds exhibited a profile of increasing inflammation and immune functional categories similar to the colonized/failed wounds. Interestingly, the non-colonized/healed wound profile for inflammation and immune functional categories was more static, either remaining flat or only slightly increasing over time.

There are several limitations to this study. First due to the nature of this cohort and the technologies used there is a limited number of samples. An increased sample size would allow for the validation of these results, and potentially identify other significant gene expression profile differences unable to be detected in this study. This cohort also cannot provide information about the earliest surgeries due to the logistics of intercontinental patient transport and coordination of study procedures, and therefore other cohorts would be needed to investigate the initial hemostasis processes. Further, this study was limited to bulk RNA sequencing, which provided a high-level overview of all the processes occurring from the heterogenous cell population in that tissue sample but does not allow for direct tracking of individual cell populations over time. Therefore, pairing these types of data with flow cytometry, single cell sequencing, or histological staining of the tissue, would allow for a better understanding of the changes in abundances of critical cell populations over time^32^. To expand upon this study the systemic response to single or multiple wounds needs to be investigated. It is known that having multiple complications and high injury burden including multiple extremity wounds, can influence the immune and inflammatory response systemically, and therefore the wound healing response, through free circulating, or exosome-bound, cytokines and chemokines^33^. It should be noted that in this study, patients with multiple wounds had the same outcome, which may not always be the case (i.e., a patient with one wound that healed whereas another failed). Pairing data such as these with circulating concentrations of key proteins could provide a more complete picture of the immune status of the patient.

In conclusion, this study provides information about the potential paths any large wound can follow over the course of its healing process (Fig. 5C). When wounds become colonized, the environment shifts to a hyper-inflammatory pro-immune functional response state that cannot progress to the proliferation phase. If wounds cannot mount an adequate defense, the wounds will remain in this hyper-inflammatory state and fail. If wounds can progress to the proliferation phase, a balance is needed between the immune/inflammatory processes and the proliferative programs to build ECM and collagen. If the inflammatory/immune response remains too great the wounds can still fail. Only wounds that can overcome initial pro-inflammatory environments regardless of colonization status and properly balance the proliferative processes through coordinated processes heal. Beyond providing more information to the wound healing process, these data provide novel functions and individual biomarkers (i.e., differentially expressed genes between failed vs. healed wounds) that can be further evaluated and incorporated into machine learning models to predict when to close the wound and whether or not there are early signs that the wound has a higher likelihood of failure as has been demonstrated previously^12,13, 20^. These types of investigations will lay the foundation for precision medicine tools to improve clinical outcomes in traumatic wound care for both combat casualties and in civilian trauma centers.

### Supplementary Information

Supplementary Information 1.

Supplementary Information 2.

## Data Availability

The raw datasets generated and analyzed in the current study are not publicly available due to sensitivities regarding their generation from an injured military service member cohort, but are available from the authors on reasonable request in accordance with applicable regulations and data usage agreements. For requests, please contact the corresponding author.
