# Supplementary material for: The influence of microbial colonization on inflammatory versus pro-healing trajectories in combat extremity wounds
Source: Sci Rep. 2024 Mar 4;14:5006. doi: 10.1038/s41598-024-52479-5 (PMC10912443; doi:10.1038/s41598-024-52479-5)
Supplement: Supplementary file 1 — Supplementary Information 1. [file 41598_2024_52479_MOESM1_ESM.pdf]

Supplementary Information to be published with: The influence of microbial colonization on inflammatory versus pro-healing trajectories in combat extremity wounds

Seth A. Schobel[1-3#*], Eric R. Gann[1-3#], Desiree Unselt[1-3&], Scott F. Grey[1-3], Felipe A. Lisboa[1-3], Meenu M. Upadhyay[2], Michael Rouse[1-3], Simon Tallowin[1,2,4], Nicholas A. Be[5], Xijun Zhang[6], Clifton L. Dalgard[6,7], Matthew D. Wilkerson[6,7], Milos Hauskrecht[8], Stephen F. Badylak[9,11], Ruben Zamora[9,10], Yoram Vodovotz[9,10], Benjamin K. Potter[1,2,12], Thomas A. Davis[1], Eric A. Elster[1,2,12]

[1]Department of Surgery, Uniformed Services University of the Health Sciences, Bethesda, MD, USA
[2]Uniformed Services University (USU) Surgical Critical Care Initiative (SC2i), Bethesda, MD, USA
[3]Henry M. Jackson Foundation for the Advancement of Military Medicine, Inc., Bethesda, MD, USA
[4]Academic Department of Military Surgery and Trauma, Royal Centre for Defence Medicine, Birmingham, UK
[5]Physical and Life Sciences Directorate, Lawrence Livermore National Laboratory, Livermore, CA, USA
[6]Uniformed Services University (USU) The American Genome Center (TAGC), Bethesda, MD, USA
[7]Department of Anatomy, Physiology & Genetics, Uniformed Services University of the Health Sciences, Bethesda, MD, USA
[8]Department of Computer Science, University of Pittsburgh, Pittsburgh, PA, USA
[9]Department of Surgery, University of Pittsburgh, Pittsburgh, PA, USA
[10]Center for Inflammation and Regeneration Modeling, McGowan Institute for Regenerative Medicine, University of Pittsburgh, Pittsburgh, PA, USA
[11]Department of Bioengineering, University of Pittsburgh, Pittsburgh, PA, USA
[12]Walter Reed National Military Medical Center, Bethesda, MD, USA

*Corresponding author
[#] Authors contributed equally to this work
[&]Current Address: Q^2 Solutions, Durham, NC, USA

Address correspondence to:
Seth A. Schobel, PhD
Department of Surgery
Uniformed Services University
6720B Rockledge Dr.
Suite 320
Bethesda, MD 20817-1884
seth.schobel-mchugh.ctr@usuhs.edu

Supplementary Datasets Legends.

Supplementary Dataset S1. DESeq2 results for all comparisons.

Supplementary Dataset S2. qRT-PCR normalized expression values which was normalized by gene and sample median center normalization for the comparison of expression of genes from the same tissue samples between qRT-PCR and this study.

Supplementary Dataset S3. RNASeq normalized expression values which was normalized by gene and sample median center normalization for the comparison of expression of genes from the same tissue samples between qRT-PCR and this study.

Supplementary Dataset S4. Spearman's rho correlation coefficients between normalized qRT-PCR and RNASeq expression values.

Supplementary Dataset S5. Gene ontologies enriched in DEG sets as determined by gProfiler.

Supplementary Dataset S6. Classification of enriched Gene ontologies into the 19 GO Functional Categories of interest.

Supplementary Dataset S7. Counts of the number of Gene ontologies into the 19 GO Functional Categories of interest.

Supplementary Dataset S8. Summed DESeq2 normalized count values for each GO functional category.

Supplementary Dataset S9. Proportion of immune cells predicted by quanTIseq for each sample.

Supplementary Dataset S10. Spearman's rho correlation coefficients for the GO Functional Categories vs. time or the quanTIseq cell proportions vs. time for each wound grouping.

Supplementary Dataset S11. Genes included in both Dynamic Network Analyses grouped by method of down selection.

Supplementary Dataset S12. Contributions each spearman's rho correlation coefficients for the GO Functional Categories vs. time or the quanTIseq cell proportions vs. time for each wound grouping for each principal component.

Supplementary Table S1. Description of samples used for RNA sequencing in this study. Washouts are labeled alphabetically, with the initial washout being labeled as 'A'. Colonizers and tissue CFUs are based on quantitative bacteriology.

Patient ID	Wound	Washout	Sample ID	Days Since Injury	Outcome	Colonizers	Tissue CFUs	Wound Location	AKI	Bacteremia	HO	MODS	Pneumonia	VAP	VTE
4	1	A	SC2i36	12	Failed	Yes	1.00×10^5	Calf	Yes	Yes	Yes	Yes	Yes	Yes	No
4	1	B	SC2i37	14	Failed	Yes	1.00×10^5	Calf	Yes	Yes	Yes	Yes	Yes	Yes	No
4	3	A	SC2i38	12	Failed	Yes	1.00×10^5	Thigh	Yes	Yes	Yes	Yes	Yes	Yes	No
4	3	B	SC2i39	14	Failed	Yes	1.00×10^5	Thigh	Yes	Yes	Yes	Yes	Yes	Yes	No
4	3	C	SC2i40	16	Failed	Yes	2.00×10^5	Thigh	Yes	Yes	Yes	Yes	Yes	Yes	No
6	1	A	SC2i8	3	Healed	No	0	Forearm	No	No	No	No	No	No	No
6	1	E	SC2i9	13	Healed	No	0	Forearm	No	No	No	No	No	No	No
8	1	A	SC2i1	5	Healed	Yes	1.00×10^5	Forearm	No	No	No	No	No	No	No
8	1	B	SC2i2	7	Healed	Yes	4.00×10^3	Forearm	No	No	No	No	No	No	No
8	1	C	SC2i3	10	Healed	Yes	9.67×10^3	Forearm	No	No	No	No	No	No	No
11	1	A	SC2i4	6	Healed	No	0	Calf	No	No	Yes	No	No	No	No
11	1	B	SC2i5	7	Healed	No	0	Calf	No	No	Yes	No	No	No	No
11	1	D	SC2i6	11	Healed	Yes	2.60×10^4	Calf	No	No	Yes	No	No	No	No
11	1	F	SC2i7	15	Healed	Yes	1.66×10^5	Calf	No	No	Yes	No	No	No	No
12	1	A	SC2i14	6	Healed	No	0	Thigh	Yes	Yes	Yes	No	Yes	No	No
12	1	B	SC2i15	8	Healed	No	0	Thigh	Yes	Yes	Yes	No	Yes	No	No
12	1	C	SC2i16	10	Healed	No	0	Thigh	Yes	Yes	Yes	No	Yes	No	No
12	1	D	SC2i17	12	Healed	Yes	2.00×10^4	Thigh	Yes	Yes	Yes	No	Yes	No	No
16	3	A	SC2i61	5	Failed	No	0	Calf	Yes	Yes	No	No	No	No	No
16	3	B	SC2i62	7	Failed	No	0	Calf	Yes	Yes	No	No	No	No	No
19	1	A	SC2i10	4	Healed	Yes	1.00×10^7	Calf	No	No	Yes	No	No	No	No
19	1	B	SC2i11	6	Healed	Yes	3.00×10^6	Calf	No	No	Yes	No	No	No	No
19	1	C	SC2i12	8	Healed	No	0	Calf	No	No	Yes	No	No	No	No

19	1	F	SC2i13	18	Healed	No	0	Calf	No	No	Yes	No	No	No	No
20	1	A	SC2i21	4	Healed	Yes	8.00×10^6	Thigh	Yes	No	Yes	No	Yes	Yes	No
20	1	B	SC2i22	6	Healed	Yes	8.00×10^5	Thigh	Yes	No	Yes	No	Yes	Yes	No
20	1	D	SC2i23	12	Healed	No	0	Thigh	Yes	No	Yes	No	Yes	Yes	No
20	1	J	SC2i24	30	Healed	No	0	Thigh	Yes	No	Yes	No	Yes	Yes	No
42	1	A	SC2i25	4	Healed	No	0	Thigh	No	No	No	No	No	No	No
42	1	C	SC2i26	8	Healed	No	0	Thigh	No	No	No	No	No	No	No
42	1	D	SC2i27	11	Healed	No	0	Thigh	No	No	No	No	No	No	No
42	1	E	SC2i28	13	Healed	No	0	Thigh	No	No	No	No	No	No	No
48	1	A	SC2i18	5	Healed	No	0	Calf	No	No	No	No	No	No	No
48	1	B	SC2i19	7	Healed	No	0	Calf	No	No	No	No	No	No	No
48	1	D	SC2i20	11	Healed	No	0	Calf	No	No	No	No	No	No	No
56	1	A	SC2i41	7	Failed	No	0	Calf	No	No	No	No	No	No	No
56	1	B	SC2i42	9	Failed	No	0	Calf	No	No	No	No	No	No	No
56	1	C	SC2i43	12	Failed	No	0	Calf	No	No	No	No	No	No	No
56	1	D	SC2i44	14	Failed	No	0	Calf	No	No	No	No	No	No	No
64	2	A	SC2i29	7	Healed	No	0	Thigh	No	Yes	Yes	No	No	No	Yes
66	1	A	SC2i59	5	Failed	No	0	Leg	No	No	No	No	No	No	No
66	1	C	SC2i60	9	Failed	No	0	Leg	No	No	No	No	No	No	No
66	2	A	SC2i55	5	Failed	No	0	Leg	No	No	No	No	No	No	No
66	2	B	SC2i56	7	Failed	No	0	Leg	No	No	No	No	No	No	No
66	2	C	SC2i57	9	Failed	No	0	Leg	No	No	No	No	No	No	No
66	2	D	SC2i58	11	Failed	No	0	Leg	No	No	No	No	No	No	No
68	1	A	SC2i66	4	Failed	No	0	Leg	No	Yes	No	No	No	No	No
68	1	B	SC2i67	6	Failed	Yes	4.70×10^7	Leg	No	Yes	No	No	No	No	No
69	1	A	SC2i52	4	Failed	No	0	Leg	No	No	No	No	No	No	No
69	1	B	SC2i53	6	Failed	Yes	1.57×10^7	Leg	No	No	No	No	No	No	No
69	2	A	SC2i63	4	Failed	No	0	Leg	No	No	No	No	No	No	No
69	2	B	SC2i64	6	Failed	Yes	1.50×10^7	Leg	No	No	No	No	No	No	No

69	2	C	SC2i65	8	Failed	No	0	Leg	No	No	No	No	No	No	No
71	1	A	SC2i45	6	Failed	No	0	Leg	No	No	No	No	No	No	Yes
71	1	B	SC2i46	8	Failed	Yes	1.00×10^8	Leg	No	No	No	No	No	No	Yes
71	1	C	SC2i47	11	Failed	No	0	Leg	No	No	No	No	No	No	Yes
71	1	D	SC2i48	13	Failed	No	0	Leg	No	No	No	No	No	No	Yes
72	1	A	SC2i49	5	Failed	No	0	Leg	No	No	No	No	No	No	No
72	1	B	SC2i50	7	Failed	No	0	Leg	No	No	No	No	No	No	No
72	1	C	SC2i51	10	Failed	No	0	Leg	No	No	No	No	No	No	No
73	2	B	SC2i32	13	Healed	No	0	Thigh	Yes	Yes	Yes	Yes	Yes	Yes	No
73	2	D	SC2i33	18	Healed	No	0	Thigh	Yes	Yes	Yes	Yes	Yes	Yes	No
73	2	E	SC2i34	21	Healed	No	0	Thigh	Yes	Yes	Yes	Yes	Yes	Yes	No
73	2	G	SC2i35	25	Healed	No	0	Thigh	Yes	Yes	Yes	Yes	Yes	Yes	No

Supplementary Table S2. Number of GO terms enriched from the differentially expressed genes sets for the comparison of wound outcome and colonization status determined by gProfiler. Both comparisons are broken into various subsets of the entire dataset including the timepoints and colonization status (for the wound outcome comparisons) and wound outcome (for colonization status). Dashed lines indicate where no comparison was performed.

Comparison: Wound Outcome: Failed v. Healed Wounds						
Wound Subset	All wounds		Colonized wounds		Non-colonized wounds	
Time Groupings	Upregulated Gene Set	Downregulated Gene Set	Upregulated Gene Set	Downregulated Gene Set	Upregulated Gene Set	Downregulated Gene Set
All Samples	0	0	0	282	0	0
Days 3 – 5	0	0	-	-	0	0
Day 6	0	0	290	496	1	41
Day 7	0	0	-	-	0	17
Days 8 – 10	0	0	-	-	0	0
Days 11 – 12	14	0	220	192	5	2
Days 13 – 16	0	0	570	192	0	6
Days 17+	-	-	-	-	-	-

Colonization Status: Colonized v. Non-colonized Wounds						
Wound Subset	All wounds		Healed wounds		Failed wounds	
Time Groupings	Upregulated Gene Set	Downregulated Gene Set	Upregulated Gene Set	Downregulated Gene Set	Upregulated Gene Set	Downregulated Gene Set
All Samples	238	43	0	0	430	34
Days 3 – 5	0	0	0	0	-	-
Day 6	0	0	140	318	0	0
Day 7	0	234	8	406	-	-
Days 8 – 10	0	0	0	0	0	0
Days 11 – 12	291	94	35	2	65	248
Days 13 – 16	92	78	0	42	285	34
Days 17+	-	-	-	-	-	-

Supplementary Table S3. Number of connections in the Dynamic Network Analysis (DyNA) for both the unbiased gene down-selection and the gene down-selection using KEGG and Reactome [29-30].

Number of RNA sequencing libraries by Wound Outcome												
**Number of Samples**	**D4**	**D5**	**D6**	**D7**	**D8**	**D9**	**D10**	**D11**	**D12**	**D13**	**D14**	**D18**
**Healed**	3	2	4	4	3	-	2	3	2	3	-	2
**Failed**	2	3	3	4	2	2	-	2	3	-	3	-
**Number of Connections for the Unbiased Gene Down-selection in Healed Wounds**												
**Day Comparison**	**D4-d5**	**D5-d6**	**D6-d7**	**D7-d8**	**D8-d10**	**D10-D11**	**D11-D12**	**D12-D13**	**D13-D18**		**Total**	
**Total**	91	37	129	141	173	234	184	194	315		1498	
**Positive**	77	37	129	141	171	228	169	186	314		1452	
**Negative**	14	0	0	0	2	6	15	8	1		46	
**Number of Connections for the Unbiased Gene Down-selection in Failed Wounds**												
**Day Comparison**	**D4-D5**	**D5-D6**	**D6-D7**	**D7-D8**	**D8-D9**	**D9-D11**	**D11-D12**	**D12-D14**			**Total**	
**Total**	18	27	24	33	178	159	184	243			866	
**Positive**	17	27	24	32	166	155	183	243			847	
**Negative**	1	0	0	1	12	4	1	0			19	
**Number of Connections for the KEGG/Reactome Gene Down-selection in Healed Wounds**												
**Day Comparison**	**D4-d5**	**D5-d6**	**D6-d7**	**D7-d8**	**D8-d10**	**D10-D11**	**D11-D12**	**D12-D13**	**D13-D18**		**Total**	
**Total**	74	28	10	61	265	63	41	101	142		785	
**Positive**	41	28	10	57	247	34	24	81	100		622	
**Negative**	33	0	0	4	18	29	17	20	42		163	
**Number of Connections for the KEGG/Reactome Gene Down-selection in Failed Wounds**												
**Day Comparison**	**D4-D5**	**D5-D6**	**D6-D7**	**D7-D8**	**D8-D9**	**D9-D11**	**D11-D12**	**D12-D14**			**Total**	
**Total**	10	4	6	24	122	133	98	24			421	
**Positive**	8	4	6	18	79	100	74	18			307	
**Negative**	2	0	0	6	43	33	24	6			114	

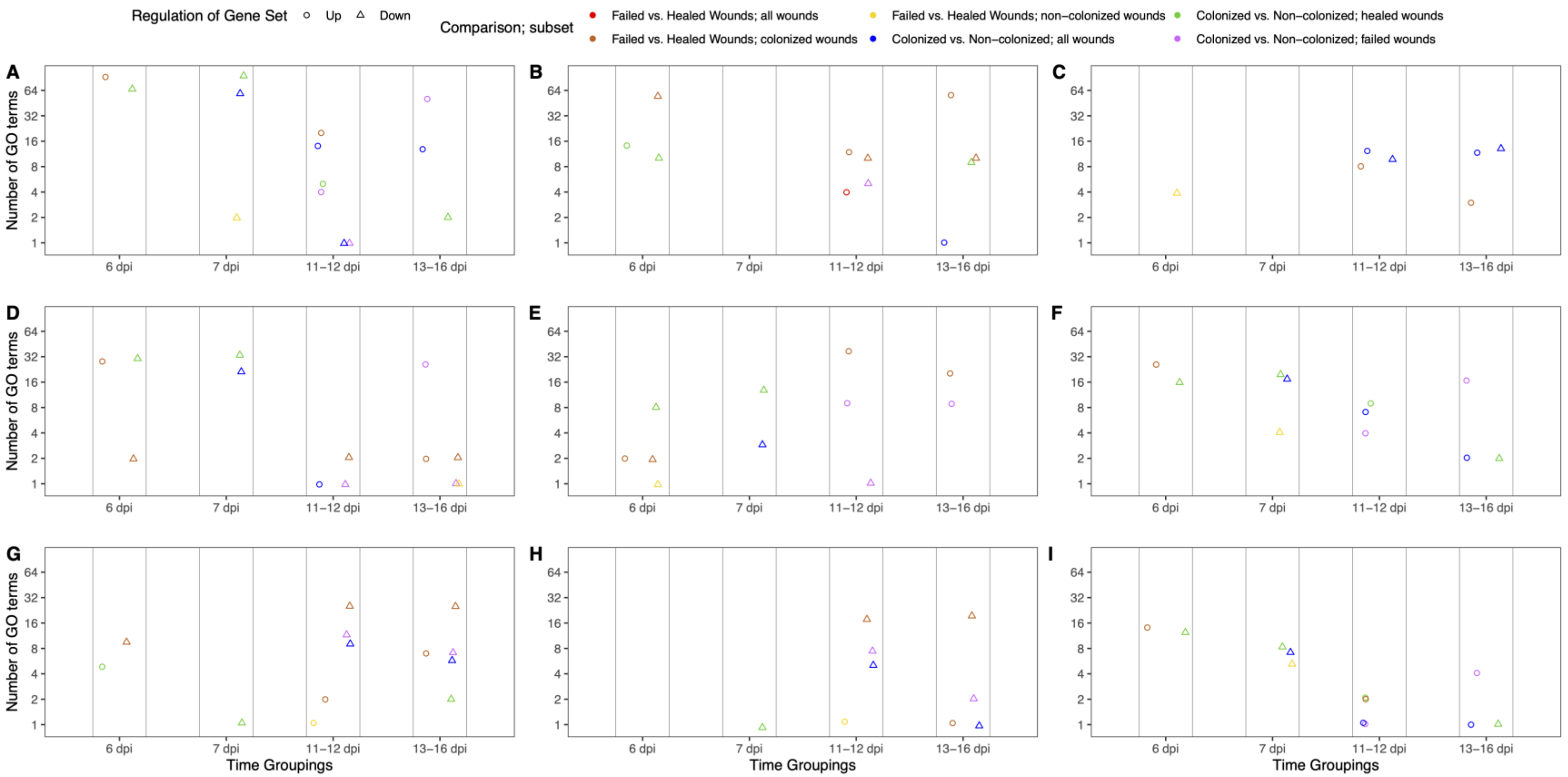

Supplementary Figure S1. Number of enriched GO terms in each DEG set for GO Functional Categories found in 10 or more DEG sets. Number of GO terms found in A) Muscle functions; B) Cytokine functions; C) Translation functions; D) Calcium functions; E) Metal ion functions; F) Actin functions, G) ECM functions, H) Collagen functions, and I) Myosin functions. GO term counts are separated by the comparison performed and the regulation of the DEG set with upregulated being denoted by circles and the downregulated DEG set being denoted by triangles.

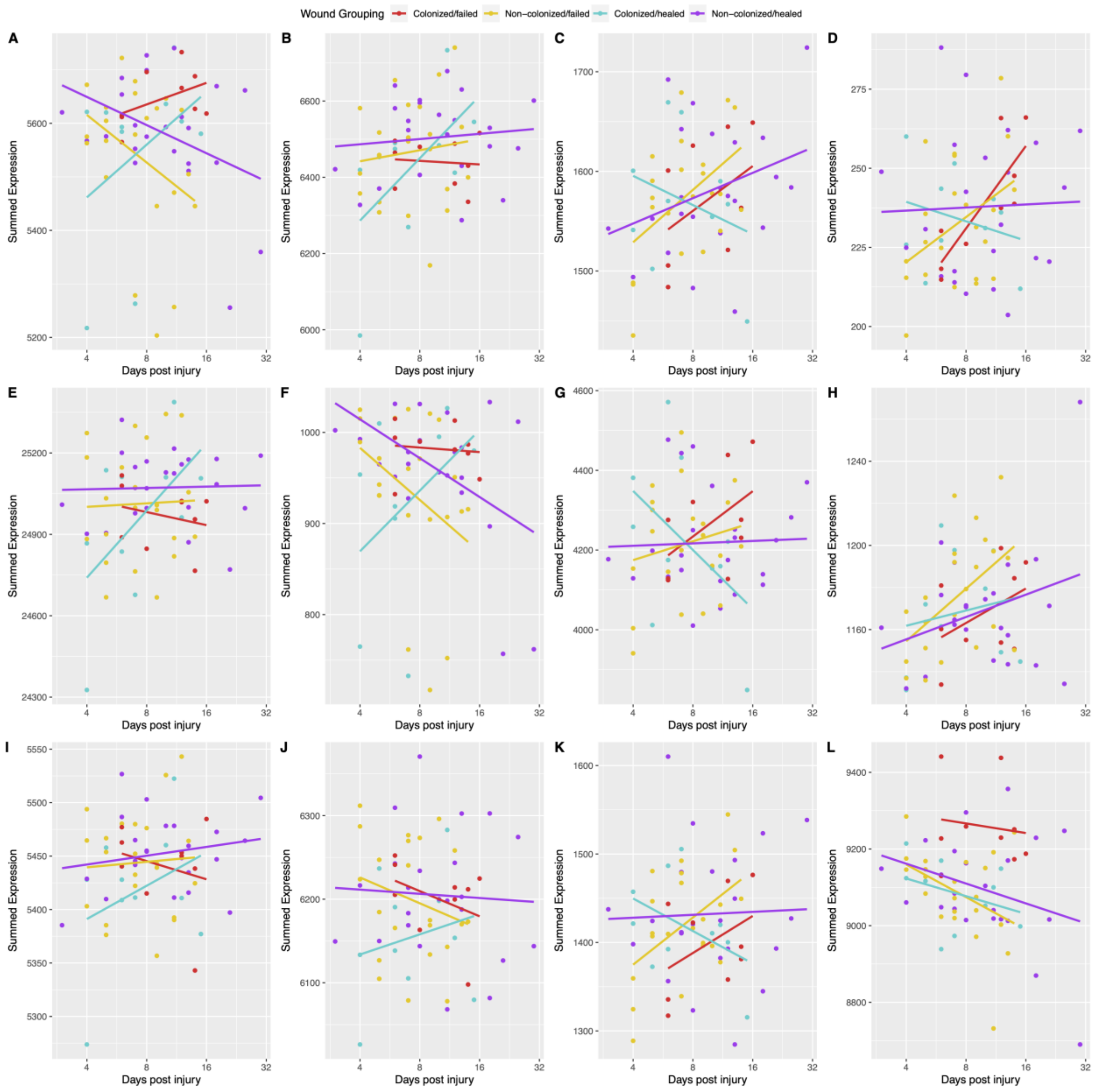

Supplementary Figure S2. Expression profiles of GO functional categories over time reveal differences in function trajectories based on wound group. The expression profiles pertain to the following GO functional categories A) Actin, B) Calcium, C) Leukocytes, D) Lymphocytes, E) Metal Ions, F) Myosin, G) Neutrophils, H) Oxygen Transport / Hemoglobin, I) Peptidases/Proteases, J) Redox, K) T cells, and L) Translation. Each point represents the summed expression of all genes within that functional category in a particular transcriptomic library. Gene sets are described in Supplemental Dataset S8. The lines represent linear models generated comparing summed expression versus time for each wound group. Wound groups are denoted by color: with cyan being for colonized/healed wounds, purple being for non-colonized/healed wounds, red being for colonized/failed wounds, and yellow being for non-colonized/failed wounds.

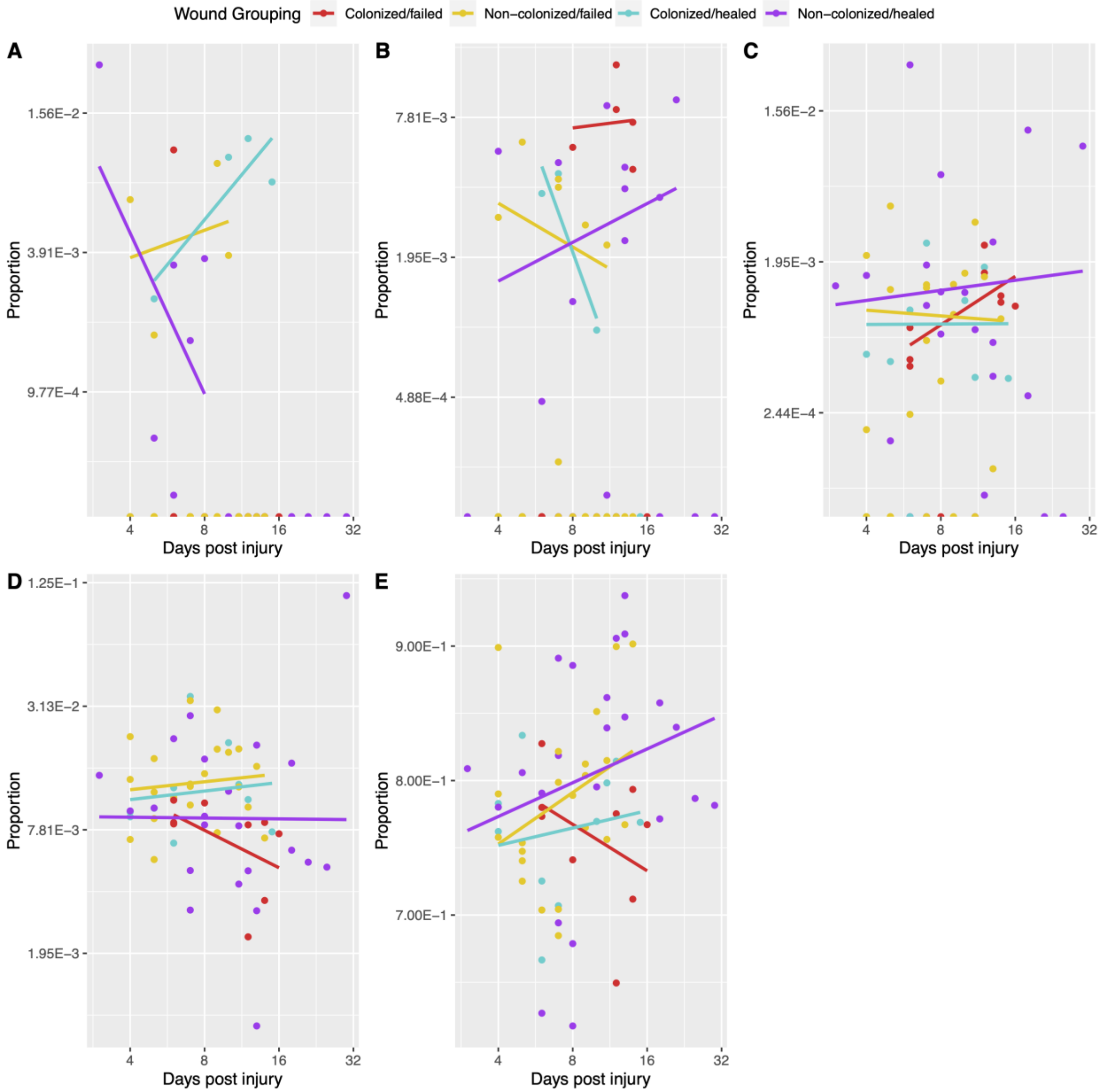

Supplementary Figure S3. Proportions of each immune cell type predicted by quanTIseq for each sample. Predicted proportions of immune cell types over time reveal differences based on wound group. The proportion of immune cells were predicted using quanTIseq and the proportions of the following are shown: of of A) Dendritic cells, B) CD4 T cells, C) CD8 T cells, D) Regulatory T cells, and E) Other not-classified cells. Each point represents the predicted proportion of that cell type in a particular transcriptomic library. Predicted proportions are described in Supplemental Dataset S9. The lines represent linear models generated comparing predicted proportions versus time for each wound group. Wound groups are denoted by color: with cyan being for colonized/healed wounds, purple being for non-colonized/healed wounds, red being for colonized/failed wounds, and yellow being for non-colonized/failed wounds.

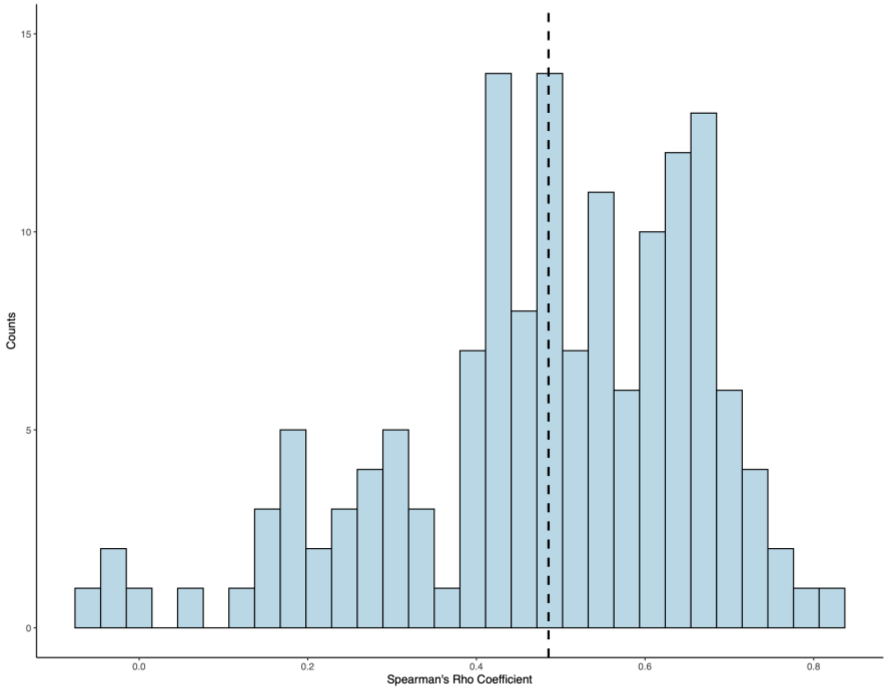

Supplementary Figure S4. Distribution of Spearman's rho coefficients for correlations between gene expression values determined previously by qRT-PCR, and by RNA-seq in this study. The dashed vertical line represents the mean of all coefficients.
